# RETRACTION: Fatty Acid Synthase Contributes to Epithelial‐Mesenchymal Transition and Invasion of Salivary Adenoid Cystic Carcinoma Through PRRX1/Wnt/β‐Catenin Pathway

**DOI:** 10.1111/jcmm.71042

**Published:** 2026-02-03

## Abstract

**RETRACTION**: ZhangW.‐L. , WangS.‐S. , JiangY.‐P. , LiuY. , YuX.‐H. , WuJ.‐B. , WangK. , PangX. , LiaoP. , LiangX.‐H. , and TangY.‐L. , “Fatty Acid Synthase Contributes to Epithelial‐Mesenchymal Transition and Invasion of Salivary Adenoid Cystic Carcinoma Through PRRX1/Wnt/β‐Catenin Pathway,” Journal of Cellular and Molecular Medicine 24, no. 19 (2020): 11465–11476, 10.1111/jcmm.15760.32820613 PMC7576276

The above article, published online on 20 August 2020 in Wiley Online Library (wileyonlinelibrary.com) has been retracted by agreement between the journal Editor‐in‐Chief, Stefan N. Constantinescu; The Foundation for Cellular and Molecular Medicine; and John Wiley and Sons Ltd. The retraction has been agreed due to concerns raised by third parties. Specifically, instances of image duplication have been identified within Figures 3A and S1. The authors have acknowledged the issues, explaining that they resulted from inaccuracies during figure assembly in manuscript preparation, and have provided the corrected data. However, further post‐publication review revealed that the article lacks critical details necessary for reproducing and interpreting the findings, including the sequences for FASN‐shRNA, FASN overexpression, PRRX1 overexpression, and their respective negative controls, as well as the absence of a legend for Figure S1. Finally, the article does not sufficiently reference relevant prior literature related to salivary adenoid cystic carcinoma in support of the study's rationale and to contextualize the study's findings, and some cited references have since been retracted, leaving related claims unsupported. Accordingly, the article has been retracted as the editors no longer consider the article's conclusions to be reliable. The authors disagree with the retraction decision.

**RETRACTION**: W.‐L. Zhang , S.‐S. Wang , Y.‐P. Jiang , Y. Liu , X.‐H. Yu , J.‐B. Wu , K. Wang , X. Pang , P. Liao , X.‐H. Liang , and Y.‐L. Tang , “Fatty Acid Synthase Contributes to Epithelial‐Mesenchymal Transition and Invasion of Salivary Adenoid Cystic Carcinoma Through PRRX1/Wnt/β‐Catenin Pathway,” Journal of Cellular and Molecular Medicine 24, no. 19 (2020): 11465–11476, 10.1111/jcmm.15760.32820613 PMC7576276

The above article, published online on 20 August 2020 in Wiley Online Library (wileyonlinelibrary.com) has been retracted by agreement between the journal Editor‐in‐Chief, Stefan N. Constantinescu; The Foundation for Cellular and Molecular Medicine; and John Wiley and Sons Ltd. The retraction has been agreed due to concerns raised by third parties. Specifically, instances of image duplication have been identified within Figures 3A and S1. The authors have acknowledged the issues, explaining that they resulted from inaccuracies during figure assembly in manuscript preparation, and have provided the corrected data. However, further post‐publication review revealed that the article lacks critical details necessary for reproducing and interpreting the findings, including the sequences for FASN‐shRNA, FASN overexpression, PRRX1 overexpression, and their respective negative controls, as well as the absence of a legend for Figure S1. Finally, the article does not sufficiently reference relevant prior literature related to salivary adenoid cystic carcinoma in support of the study's rationale and to contextualize the study's findings, and some cited references have since been retracted, leaving related claims unsupported. Accordingly, the article has been retracted as the editors no longer consider the article's conclusions to be reliable. The authors disagree with the retraction decision.

